# Malignant salivary gland tumours of the head and neck region: a single institutions review

**DOI:** 10.11604/pamj.2015.20.121.3458

**Published:** 2015-02-12

**Authors:** Ahmed Oluwatoyin Lawal, Akinyele Olumuyiwa Adisa, Bamidele Kolude, Bukola Folasade Adeyemi

**Affiliations:** 1 Department of Oral Pathology, College of Medicine, University of Ibadan, Nigeria

**Keywords:** Malignant salivary gland tumour, adenoid cystic carcinoma, mucoepidermoid carcinoma, polymorphous low-grade adenocarcinoma

## Abstract

**Introduction:**

Malignant salivary gland tumours (MSGTs) comprise about 3% of all head and neck cancers; they demonstrate an unpredictable clinical course. The purpose of this study is to review MSGTs seen at a tertiary Health centre, and compare findings with those of previous studies.

**Methods:**

The records of the Department of Oral Pathology and the Department of Pathology, University College Hospital Ibadan were reviewed over a 19 year period and lesions diagnosed as MSGTs according to 2005 WHO histological classification were analysed for age, gender and site using SPSS for Windows (version 20.0; SPSS Inc. Chicago, IL).

**Results:**

MSGTs were more common in males (55.2%) than females (44.8%). The mean age of was 47.9 (±17.0) years and peak age was the fifth decade. The parotid gland was the commonest site with 62 (28.1%) cases. The palate was the commonest intraoral site with 61(27.6%). The nose with 19 (8.6%) was the commonest minor extra-oral site.

**Conclusion:**

The findings were essentially similar to reports from Europe and America. Adenoid Cystic Carcinoma was the most common MSGT in this series. A high proportion of salivary gland tumours in sublingual gland were malignant. The reason(s) for high proportion of MSGTs in sublingual glands requires further investigation.

## Introduction

Malignant salivary gland tumours (MSGTs) comprise about 3% of all head and neck cancers and demonstrate an unpredictable clinical course marked by frequent locoregional failure and distant metastasis, often occurring years after diagnosis [1]. The treatment of MSGTs is often challenging mainly due to their unpredictable and varied biologic behaviour and their prolonged risk of recurrence [2]. Although, the aetiology of MSGTs is essentially unknown, high or prolonged doses of radiation to the head and neck have been shown to be risk factors for salivary gland cancers in previous studies [3]. Other reports have suggested that exposure to silica, vegetable depleted and cholesterol rich diets may be additional risk factors for development of salivary gland malignancies [3]. In addition, reports from several studies have shown that a history of prior cancer, especially those related with ultraviolet radiation, immunosuppression and Epstein-Barr virus were found to be associated with an increased occurrence of MSGTs [4].

The results of the demography of salivary gland tumours from previous studies have been somewhat varied. Although, most authors reported that MSGTs are less commonly seen than their benign counterparts [5–7], Ladeinde et al, [8] reported that MSGTs represented 60.8% of SGTs while benign SGTs accounted for 40.2% of their cases. Also, the controversy of which lesion is the commonest MSGT is not entirely resolved as many studies have reported that adenoid cystic carcinoma (ACC) was the most common MSGT [6, 7], while some others reported that mucoepidermoid carcinoma (MEC) was the most common MSGT [9, 10]. Although, many studies [6, 9, 11–13] have examined the demography of salivary gland tumours, studies examining MSGTs as a group are rare [14, 15]. The purpose of this study was to review MSGTs seen at tertiary Health Centre and compare findings with those of previous studies.

## Methods

The records of the Department of Oral Pathology and the Department of Pathology, University College Hospital Ibadan were reviewed over a 19 year period and lesions diagnosed as MSGTs according 2005 WHO histological classification were analysed for age, gender and site using SPSS for Windows (version 20.0; SPSS Inc. Chicago, IL). Ethical approval for the study was obtained from the Oyo State Research Ethical Review Committee (AD 13/479/435).

## Results

A total of 221 MSGTs representing 53.5% of the total number of salivary gland tumours (SGTs) were seen over the study period. MSGTs were more commonly seen in males (55.2%) than females (44.8%) with a male to female ratio of 1.2:1. The mean age of occurrence of MSGTs was 47.9 (±17.0) years (range = 8 to 98 years) and peak age incidence was in the fifth decade of life. The mean age for males was 46.70 (±17.6) years while for females it was 49.36 (±16.1) years. MSGTs occurred more above the age of 40 years with 70.2% of cases seen in ages forty and above while only 29.8% occurred below the age of forty.

Table 1 shows that ACC with 93 cases was the most common MSGT accounting for 42.1% of total number of MSGTs in this study. MEC with 27.6% of cases was the second most common MSGT while adenocarcinoma not otherwise specified (Adeno Ca NOS) with 13.6% of cases was the third most common MSGT. Other MSGTs such as acinic cell carcinoma, polymorphous low grade adenocarcinoma (PLGA), carcinoma ex-pleomorphic adenoma and cystadenocarcinoma were occasionally seen all accounting for 17% of total number of MSGTs.

**Table 1:** Histologic types of malignant salivary gland tumours

SGTs	N (%)
adenoid cystic carcinoma	93(42.1)
mucoepidermoid carcinoma	61(27.6)
Adenocarcinoma not otherwise specified	30(13.6)
Polymorphous low-grade adenocarcima	7(3.2)
papillary cystic adenocarcinoma	7(3.2)
acinic cell carcinoma	6(2.7)
carcinoma –ex-pleomorphic adenoma	5(2.3)
Basal cell adenocarcinoma	3(1.4)
Squamous cell carcinoma	3(1.4)
Mucinous adenocarcinoma	2(0.9)
Salivary duct carcinoma	2(0.9)
Oncocytic carcinoma	1(0.5)
Sebaceous carcinoma	1(0.5)
Total	221(100.0)

Eighty four cases (38%) of MSGTs were seen in major salivary glands while 83 (37.8%) and 53 (24%) cases were seen in minor and minor extra oral (ectopic/accessory glands) salivary glands respectively. The parotid gland was the commonest site of occurrence with 62 (28.1%) cases while the submandibular and sublingual glands accounted for only 16 (7.2%) and 5 (7.2%) cases respectively. The palate was the commonest intraoral site with 61 (27.6%) cases followed by the mandible with 11 (5%) cases and the buccal mucosa with 10 (4.5%) cases. The nose and the antrum which accounted for 19 (8.6%) and 16 (7.2%) cases respectively were the commonest sites for MSGTs in ectopic/accessory glands salivary glands.

MSGTs tumours accounted for 36.3% of all parotid gland salivary gland tumours, while they are 32.7% of salivary gland tumours in the submandibular gland and 71.4% of salivary gland tumours in sublingual gland. Table 2 shows an overview of age, site and sex distribution of the most commonly seen MSGTs in this study.

**Table 2:** Sex, site and age distribution of the most common malignant salivary gland tumours

Diagnosis	Sex			Age			Site N (%)			
	N (%)	M (%)	F (%)	Mean age	Peak age	Parotid	Subm gland	Subli gland	minor	ectopic
ACC	93(42.1)	52(55.9)	41(44.1)	49.9	60-69	20(21.5)	3(3.2)	1(1.1)	50(53.8)	19(20.4)
MEC	61(27.6)	34(55.7)	27(44.3)	42.9	50-59	24(39.3)	10(16.4)	-	17(27.9)	10(16.4)
Adeno Ca NOS	30(13.6)	14(46.7)	16(53.3)	51.4	40-49	5(16.7)	1(3.3)	1(3.3)	8(26.7)	15(50.0)
PCAC	7(3.2)	5(71.4.)	2(28.6.)	41.3	10-19 30-39	1(14.3)	0(0.0)	0(0.0)	0(0.0)	6(85.7)
Ca ex PA	5(2.3)	3(60.0)	2(40.0)	48.6	70-79	3(60.0)	0(0.0)	0(0.0)	1(20.0)	1(20.0)
PLGA	7(3.2)	3(42.9)	4(57.1)	55.0	60-69 70-79	1(14.3)	1(14.3)	1(14.3)	4(57.1)	1(14.3)
AcCa	6(2.7)	3(50.0)	3(50.0)	45.5	20-29	3(50.0)	0(0.0)	2(33.3)	1(16.7)	0(0.0)

Subm = submandibular gland, subli = sublingual gland, M = male, F = female, ACC = adenoid cystic carcinoma, MEC = mucoepidermoid carcinoma, Adeno Ca NOS = adenocarcinoma not otherwise specified, Ca ex Pa = carcinoma –ex-pleomorphic adenoma, PLGA = polymorphous low grade adenocarcinoma, AcCa = acinic cell Carcinoma, PCAC= papillary cystic adenocarcinoma

## Discussion

The wide age range (8 to 98 years) gotten from this study was in agreement with those available from literature [1, 10, 12]. The mean age of 47.9 years gotten from this study was lower than those of previous studies from Brazil [12] and USA [1] who got mean ages of 55 years and 58.6 years respectively and this difference may be due to the lower life expectancy in Nigeria [16]. The gender distribution of MSGTs from previous studies has been rather conflicting. Some western studies indicated that there was no sex predilection for MSGTs [17]. In contrast; studies from Zimbabwe [9], Mexico [10] and Brazil [12] all reported a male preponderance for MSGTs which was in agreement with our finding. However, studies in negroid populations in the United States and in Africa, reported a female preponderance for MSGTs [14], though these studies reviewed only intraoral minor salivary gland tumours.

The finding that MSGTs occurred more in major salivary glands with the parotid gland being the most common site of occurrence is in agreement with most previous studies [12, 13, 17]. Although, MSGTs in the sublingual gland were rare in this series, 71.4% of all salivary gland tumours in sublingual gland were malignant. This finding was in conformity with previous studies which reported that 80-90% [18], 84% [19] and 100% [20] of salivary gland tumours in the sublingual gland were malignant. The reason for high proportion of MSGTs in the sublingual gland compared to their benign counterparts is not clear but the fact that the sublingual gland may be readily accessible to dissolved and pooled carcinogens because of its many ductal openings may be contributory.

ACC with 42.1% of cases was the most common MSGT seen. Many previous studies have reported MEC to be the most common MSGT [1, 10, 13, 17]. On the contrary, Chidzonga et al [9] from Zimbabwe, Al-Khateeb et al [6] from Jordan and Ostman et al [14] from Sweden all found ACC to be the most common MSGT. Some authors suggested that the fact that PLGA was formerly considered as ACC might have accounted for its high prevalence in some of these studies [12]. However, we believe that the rarity of PLGA as previously reported [6] may not have significantly affected the reported incidence of ACC and it is more likely that geographical variations in the incidence of ACC and MEC will better explain these conflicting reports on ACC and MEC.

ACC with a slight male predilection had a mean age of 49.9 years and peak age incidence in the seventh decade of life. These findings were essentially similar to those of Sur et al in South –Africa who got a mean age of 52 years and an equal male: female occurrence [21]. However, some studies from Brazil [22], Jordan [6] and Mexico [10] all reported a female preponderance. Fifty per cent of ACC were found in minor salivary glands with 38.7% occurring in the palate. Many previous studies agree with this finding [12, 17], although, Otoh et al [13] reported 100% of ACC in the palate and Al-Khateeb et al [6] reported a lower percentage of 25% of ACC occurring in the palate. However, a few studies have reported that ACC was more common in the parotid and submandibular glands than the minor glands [12]. MEC which was the second most common MSGT in this study has been reported to constitute about 29% to 34% of MSGTs from previous studies [23, 24] which was in conformity with our finding. Although, our finding of a peak age incidence of sixth decade was similar to that obtained by Otoh et al [13] from a study in North-East Nigeria, our mean age of 42.9 was higher than theirs. Other studies by Ledesma-Montes from Mexico [10], Al-Khateeb from Jordan [6], Rapidis et al [23] from Greece and Kokemueller et al [25] from Germany reported mean ages of 37 years, 38 years, 56.7 years and 50 years respectively. This differential between the mean ages from European studies and those from Africa and Mexico may be related to the higher life expectancy in the developed countries.

The anatomical distribution of MEC was similar to previous findings with predilection for the major salivary glands and 39.3% of cases were seen in the parotid gland. Brandwein et al [26] in a study in New York reported that 42% of MEC in their series were seen in the parotid gland while only 15% occurred in the palate. Similarly, Rapidis [23] reported that 66.6% of MEC were found in major salivary glands while 50% of cases were found in the parotid gland. Other authors [12, 27] confirmed the predilection of MEC for the major salivary glands especially the parotid gland, though no reason has been adduced for this finding. MEC was more commonly seen in males as reported by Ito et al [12] and Ostman et al [14] but some other studies reported a female preponderance [12, 28].

Adenocarcinoma, NOS is a malignant salivary gland tumour that exhibits ductal differentiation but lacks any of the histomorphologic features that characterize the other defined types of salivary carcinoma [29]. The modifying term “not otherwise specified” is included to distinguish it from many other epithelial salivary gland malignancies that are also adenocarcinomas. The finding from this study that Adenocarcinoma, NOS was the third most common salivary gland malignancy was corroborated by studies from Mexico [10] and China [17]. Although, reports from the Armed Forces Institute of Pathology (AFIP), found Adenocarcinoma, NOS to be second in frequency only to MEC among MSGTs, their finding of Adenocarcinoma, NOS representing 17% of salivary gland malignancies was similar to our finding of 13.6% [29]. However, reports from other African studies show that Adenocarcinoma NOS was relatively rare with Otoh et al [13] from Nigeria and Chidzonga et al [9] from Zimbabwe reporting frequencies of 2.5% and 3.9% respectively. The lower frequencies obtained in their studies may be attributed to their small sample size and the fact that up to 50% of cases in our series were found in ectopic sites which were not considered in their studies. Our finding of a slight female predilection and mean age of 51.4 years were in agreement with previous report from AFIP, though they reported a slightly higher mean age of 58 years [29].

PLGA is a malignant epithelial tumour characterized by cytologic uniformity, morphologic diversity, an infiltrative growth pattern, and low metastatic potential [29]. PLGA was only first described simultaneously in 1983 by two groups of re-searchers under different names [30]. Batsakis et al [30] called it terminal duct carcinoma and Freedman et al named it as lobular carcinoma. Previous studies found PLGA to be the second most frequently diagnosed malignant neoplasm of the minor salivary glands, with approximately 60% of the cases located in the palate [30]. This was in tandem with our finding of 57.1% occurring in the minor salivary glands and only 14.3% seen in the parotid gland. Also, the mean age of 55 years and a female preponderance gotten from this study were similar to findings from previous reports [30].

## Conclusion

This study which reviewed MSGTs is one of the very few from the African continent and findings were essentially similar to reports from Europe and America. ACC was the most common MSGT in this series. Though few cases of MSGTs were seen in sublingual gland, a high proportion of salivary gland tumours in sublingual gland were malignant. The reason(s) for high proportion of MSGTs in sublingual glands requires further investigation.
